# Poor quality for the poor? A study of inequalities in service readiness and provider knowledge in Indonesian primary health care facilities

**DOI:** 10.1186/s12939-021-01577-1

**Published:** 2021-11-04

**Authors:** Manon Haemmerli, Timothy Powell-Jackson, Catherine Goodman, Hasbullah Thabrany, Virginia Wiseman

**Affiliations:** 1 Department of Global Health and Development, London School of Hygiene & Tropical Medicine, 15-17 Tavistock Pl, London, WC1H 9SH UK; 2 Kirby Institute, University of New South Wales, Sydney, Australia; 3 Centre for Health Economics and Policy Studies, University of Indonesia, Jakarta, Indonesia

**Keywords:** Inequalities, Quality of care, Universal health coverage, Health insurance, Indonesia

## Abstract

**Background:**

For many low and middle-income countries poor quality health care is now responsible for a greater number of deaths than insufficient access to care. This has in turn raised concerns around the *distribution* of quality of care in LMICs: do the poor have access to lower quality health care compared to the rich? The aim of this study is to investigate the extent of inequalities in the availability of quality health services across the Indonesian health system with a particular focus on differences between care delivered in the public and private sectors.

**Methods:**

Using the Indonesian Family Life Survey (wave 5, 2015), 15,877 households in 312 communities were linked with a representative sample of both public and private health facilities available in the same communities. Quality of health facilities was assessed using both a facility service readiness score and a knowledge score constructed using clinical vignettes. Ordinary least squares regression models were used to investigate the determinants of quality in public and private health facilities.

**Results:**

In both sectors, inequalities in both quality scores existed between major islands. In public facilities, inequalities in readiness scores persisted between rural and urban areas, and to a lesser extent between rich and poor communities.

**Conclusion:**

In order to reach the ambitious stated goal of reaching Universal Health Coverage in Indonesia, priority should be given to redressing current inequalities in the quality of care.

**Supplementary Information:**

The online version contains supplementary material available at 10.1186/s12939-021-01577-1.

## Background

In line with the Alma Ata declaration in 1978, health policymakers have long focused on improving access to health care, particularly in deprived areas [8]. However, disparities in health outcomes remain wide [2, 29] and it has become increasingly clear that poor quality of care stands in the way of improved access translating into better health [7]. The Lancet Global Health commission argued that a high quality health system should exhibit an “absence of disparities in the quality of health services between individuals and groups with different levels of underlying social disadvantage” [17]. However, evidence on the inequalities in quality of care remains scarce. Although a few studies have shown that poorer groups are more likely to receive lower quality care [4, 17], questions remain regarding the underlying drivers of these inequalities. Das et al. laid out three ways in which inequalities in the quality of care can arise [8]. First, inequalities can occur when health facilities located in poor communities provide worse quality compared to health facilities located in richer communities (e.g. inadequate infrastructure, unqualified providers, etc.). Secondly, inequalities can arise when individuals of higher socioeconomic status (SES) access and utilise better health services compared to poorer individuals. Travel costs and price of health services can be significant determinants of access to quality services, affecting people of varying SES differently. Finally, inequalities may arise when a health worker provides different health services based on the patients’ SES (e.g. fewer procedures, fewer diagnostic tests, smaller consultation time).

This study focuses on the first aspect by measuring the extent to which health facilities located in poor communities provide lower quality compared to health facilities located in richer communities, which we refer to as ‘inequality in the availability of quality services’. Relatively few studies have looked at this type of inequality, perhaps reflecting the rarity of having data on both quality of care and catchment population SES in the same geographical area. The available studies indicate consistent evidence that areas with low SES tend to be served by providers with lower competence [5, 9, 11, 14, 16, 18] and by facilities with limited equipment and infrastructure [25, 26].

In Indonesia, the population of nearly 276 million individuals is scattered across approximately 6000 islands and the health system is highly decentralised. Ensuring that everyone has access to quality care is a challenging goal in such a context. Recently, the World Bank conducted an assessment of a nationally representative sample of 686 Indonesian public and private primary health care facilities. This report highlights significant gaps in the readiness of primary health care facilities to deliver a basic level of quality of care [33]. While quality of care is reported to be a nationwide problem, large geographical inequalities in the quality of care have been reported. Variations in health outcomes between provinces remain significantly large: in the eastern provinces of West Papua, Papua, Central Kalimantan, Central Sulawesi, and Maluku, the maternal mortality ratio (MMR) is above 200 per 100,000 live births; but Jakarta capital city region, Jambi, West Java, Bali, and Lampung have MMRs below 100 [32]. Only one study has analysed the extent of inequalities in provider knowledge across different wealth groups [3]. They found no significant differences across wealth groups in performance for curative care, however, for prenatal care, the poor had access to health care providers with scores 5.9 percentage points higher than those of providers available to the wealthiest patients. This study, which is now more than a decade old and uses data from 1997, is no longer up to date.

So far, studies of inequalities in quality of care in Indonesia have almost exclusively focused on the gap between islands and between urban and rural areas. Additionally, most of these studies have focused on structural aspects of quality, with limited consideration of clinical processes of care. Given Indonesia’s significant reforms designed to ensure financial protection to all members of the public, it is essential that progress in terms of equitable availability of high quality care is assessed. The aim of this study is to understand the extent of inequalities in quality of care beyond the provincial and rural/urban divide, and to present evidence on socio-economic inequalities in the availability of quality care at public and private primary health care facilities in Indonesia.

## Methods

### Policy context in Indonesia

In 2014, Indonesia took a significant step towards Universal Health Coverage by implementing a comprehensive national social health insurance scheme known as the Jaminan Kesehatan Nasional (JKN) to address growing disparities in health-care and to make comprehensive health care available to its entire population [22]. The JKN brings together all major existing health insurance schemes under a single agency - the Social Security Agency for Health (BPJS Health) - which was made mandatory for all Indonesians. JKN members must register with a primary care provider within 3 months of becoming a member, and can choose to register with either a public or a private provider contracted with BPJS-Health. Indonesia has made significant progress in JKN coverage, which has increased from 46.5% of the population in 2014 to 85% in March 2021, representing 223 million people (https://bpjs-kesehatan.go.id). This makes the JKN one of the biggest single payer health systems in the world.

In Indonesia’s public sector, primary health centres or “puskesmas” form the backbone of the system, with catchment areas of 25,000–30,000 individuals. The number of puskesmas has been gradually increasing since the late 1960s as the central element in the government’s efforts to improve access to primary health care. In 2014, there were 9731 puskesmas, which provide a set of mandatory services and tasks that include curative, rehabilitative, preventive and promotive services delivered within the facility and through outreach programmes [35]. Puskesmas are linked to a network of auxiliary health centres, called “pustu”, that provide community outreach services in remote areas.

The role of the private sector is important in Indonesia; two thirds of outpatient care and about one-half of inpatient care are provided by the private sector [33]. The private primary health care sector is diverse, and no systematic information is available at the central level on their number and distribution. Delivery of primary health care is provided in the great majority by private clinics, private physicians, and private dentists. Private midwives and nurses are also permitted to run their own clinics. Latest figures show that 42% of private clinics, 60% of private hospitals and 14% of private general practitioners are contracted with BPJS-Health to provide services to JKN patients [1].

### Data and sample

We used the Indonesian Family Life survey (IFLS) 5 in this study. The fifth wave of this survey was fielded in 2014/2015 and contains information from 16,931 households living in 312 communities (enumeration areas) from 13 provinces, and is representative of 83% of the Indonesian population. An interesting feature of the IFLS is that the household survey can be linked with a health facility survey, containing detailed information on private and public primary health providers located in the same communities. The term “community” in the IFLS refers to the primary sampling unit. We used the IFLS data to link, at the community level, information on households’ SES with information on the quality of their local primary health care facilities.

The IFLS facility survey contains data on 959 primary public and 2544 private health care providers in the IFLS communities. The provider survey sampling frame was drawn from information reported by households on local providers they knew about within their communities. The list was not restricted to facilities that the respondents used, thus avoiding potential biases associated with a choice-based sample. Health facilities were divided into two strata: one stratum of public primary health facilities, including health centres (puskesmas) and sub-health centres (pustu), and one stratum of private primary health facilities, including private clinics, individual practices of general practitioners (GP), and nurses/midwives practices. Within each stratum, up to five private facilities and three public facilities were selected, reflecting typically higher numbers of private providers. A description of the two surveys can be found here: https://www.rand.org/well-being/social-and-behavioral-policy/data/FLS/IFLS/ifls5.html

### Measures of quality

We used two measures of quality of care in this study: one is a supply-side readiness score used as a proxy for structural quality, and the other is a provider clinical knowledge score used to proxy clinical process quality [12].

The choice of indicators to measure supply-side readiness was informed by the Service Availability and Readiness Assessment (SARA) tool [34]. Among the many indicators collected as part of the SARA survey, the “*general service readiness”* section collects information on the potential of health facilities to provide basic health care interventions. The overlapping indicators between the IFLS provider survey and the SARA general service readiness section were identified (more than 80% of SARA indicators were found in the IFLS provider survey). The SARA indicators were then classified into five general service readiness domains (basic amenities, basic equipment, infection prevention, essential medicines and diagnostic capacity) and coded as binary variables, 1 indicating the presence of the item as reported by the provider and 0 otherwise. The full list of indicators is summarised in Appendix 1. For each domain, the percentage of items available was computed at the facility level, and the unweighted mean across the five domains was generated as an overall facility readiness score.

We developed a knowledge score using provider responses to medical vignettes, representing four different cases: an adult presenting with cough and fever, an adult presenting with diabetes, a child presenting with diarrhea and vomiting, and a pregnant woman coming for antenatal care. For each vignette, the provider who has trained in the related field and receives most of the corresponding cases in the facility was eligible to answer the questions – this meant that the provider responding to each vignette could vary within a health facility. If the facility did not provide the service corresponding to the vignette, the corresponding score was coded as a missing value. After the clinical case was described, the provider was asked what questions or activities they would ask or perform for history taking, physical examination, laboratory tests, and follow-up recommendations. Responses were either mentioned spontaneously or prompted against a prepared list of options. Not all the options were considered good practice and the correct answers were coded based on international guidelines. Details about the criteria are listed in Appendix 2. For each vignette, the percentage of correct criteria the provider mentioned without prompting was computed, and the unweighted mean across the four vignettes was generated as an overall facility knowledge score.

### Measuring community socioeconomic status

Using the IFLS household survey, the monthly household consumption was computed based on food consumption, non-food consumption, durables, education and housing expenditures, and the per capita consumption was derived by dividing total household consumption by household size. The SES of each community was computed using the mean per capita monthly consumption of households in that community. Finally, the 312 IFLS communities were divided into 5 equal SES quintiles (Q5 representing the highest SES quintile) based on their mean level of monthly household per capita consumption.

### Analysis

Using the IFLS unique community code, each health facility was linked to the corresponding community level information such as the SES quintile, the mean level of monthly household per capita consumption and type of location (urban or rural). Two main outcome variables were considered for each facility: the readiness score, and the mean knowledge score across the four vignettes. All analyses were weighted using facility sampling weights.

Descriptive numbers of facilities of each type (Puskesmas, pustus, private GP practices, private clinics and midwife/nurse practices) were presented by community SES quintile, location (rural/urban) and type of provider (JKN/non-JKN provider). Readiness and vignettes scores were computed for each facility and were summarised by facility type.

For each facility type, we examined bivariate associations between the readiness and knowledge scores, and community SES group, location, island and provider type. To harmonize the sample sizes across provinces, we recoded the province variable into larger groupings of provinces. The following islands (=grouping of provinces) were considered: Central Java (including Central Java and Yogyakarta city provinces), West Java (including Jakarta city, West Java and Banten), East Java (including East Java province only), Sumatera (including Aceh, North Sumatera, West Sumatera, South Sumatera, Lampung and Bangka Belitung Islands provinces), Lesser Sunda islands (including Bali and West Nusa Tenggara islands), Kalimantan (including South Kalimantan only) and Sulawesi (including South and West Sulawesi). To assess the extent of the inequalities in quality of care, equity gaps were computed to assess any significant difference in mean quality scores between communities belonging to Q5 (richest) and Q1 (poorest). T-tests were performed to assess any significant difference in quality scores between facilities located in rural and urban areas, as well as between facilities providing (or not) services to JKN patients.

We conducted multivariate analysis to examine differences in quality with respect to SES when controlling for other known drivers of quality, using the following linear model:

$${q}_{ij}={\beta}_0+{\beta}_1{w}_j+\gamma X+\varepsilon$$

where *q*_*ij*_ is the readiness or knowledge score of facility i in community j, *w*_*j*_ is the main explanatory variable, i.e. the SES quintile of community j, X a vector of control variables and *ε* the error term. For both readiness and knowledge scores, the model was estimated separately for public and private facilities using OLS regressions. Standard errors were clustered at the community level. Covariates included variables known to influence quality: location of the facility (rural/urban), provider type (puskesmas or pustus for the public sector, and GP practices, clinics and midwife/nurse practices for the private sector), a binary variable depending on whether the facility offered care to JKN patients, island fixed effects,^1^ and vignette dummies to control for the number and nature of the vignettes answered. In order to understand in more depth the drivers of inequality in readiness scores, the same regression model was estimated for each sub-domain of the readiness score.

## Results

The sample consisted of 2544 health facilities, among which 959 were public health facilities (671 puskesmas and 288 pustus) and 1585 were private (304 individual private practices, 195 private clinics and 1086 midwife or nurse practices). Table 1 describes how these health facilities were distributed across community SES quintiles, location (rural/urban), as well as whether these facilities provided services for JKN members. Within public health facilities, both puskesmas and pustus were equally distributed across poor and rich communities. However, puskesmas and pustus were both more likely to be located in urban areas. At the time of the survey, 97 and 88.5% of the puskesmas and pustus, respectively, were providing services for JKN patients. Within the private sector, higher level facilities (clinics and GP practices) were more likely to be found in richer areas than lower level facilities (midwife/nurse practices). Both private GP practices and clinics were also more likely to be located in urban areas, whereas midwife and nurse practices were equally distributed between urban and rural areas. Around 25% of private providers were providing services to JKN patients at the time of the survey.

**Table 1:** Descriptive statistics of sampled health facilities

	Public sector				Private sector					
	Puskesmas *N* = 671		Pustus *N* = 288		GP practices *N* = 304		Private clinics *N* = 195		Midwife/nurse practices *N* = 1086	
**Community SES quintile**	**N**	%	N	%	N	%	N	%	N	%
Q1 Poorest (mean $50)	139	21.7	49	17.4	42	15.2	9	5.5	261	28.4
Q2 Poorer (mean $62)	131	19.5	58	21.1	41	15.9	23	12.9	249	24.7
Q3 Middle (mean $75)	124	17.6	71	23.0	64	21.4	43	21.5	215	18.8
Q4 Richer (mean $91)	127	18.9	62	23.0	66	20.0	58	27.3	191	15.6
Q5 Richest (mean $142)	150	22.3	48	15.5	91	27.6	62	32.8	170	12.5
**Type of location**										
Urban	510	74.6	178	61	262	85.4	177	88.4	663	54.0
Rural	161	25.4	110	39	42	14.6	18	11.6	423	46.0
**JKN provider**										
yes	650	97.1	256	88.5	66	22.0	55	25.9	266	24.4
no	21	2.9	32	11.5	238	78.0	140	74.1	820	75.6

In Table 2, the mean readiness and knowledge scores are presented by facility type. The overall readiness score varied between 53.5% in pustus to 83.2% in puskesmas. Scores of basic amenities and standard precautions for infection prevention were overall quite high across all facility types. However, basic equipment, availability of essential medicines and diagnostic capacity scores were low. This was particularly the case in midwife/nurse practices, GP practices and pustus, where the diagnostic capacity was all below 50%. Availability of essential medicines was below 60% in all but puskesmas and private clinics. The overall level of providers’ knowledge was quite poor, with an average knowledge score below 50% for all provider types. Variation was observed across vignettes; with the curative care for children vignettes scoring the highest and the curative care for adult with diabetes vignette the lowest. Substantial variation was observed across providers as well, with puskesmas performing best on overall provider knowledge (48.8%) and midwife/nurse practices performing the worst (39.3%).

**Table 2:** Readiness and vignette scores by facility type

	Public sector		Private sector		
	Puskesmas *N* = 671	Pustus *N* = 288	GP practice *N* = 304	Private clinics N = 195	Midwife/nurse practices *N* = 1086
Basic amenities (%)	88.3	72.3	88.3	87.8	86.2
Basic equipment (%)	79.5	40.6	46.0	60.3	52.4
Standard precautions for infection prevention (%)	98.0	82.7	85.0	93.7	88.1
Diagnostic capacity (%)	69.7	14.3	18.8	35.8	20.3
Essential medicines (%)	80.7	57.7	58.5	60.9	46
**Overall readiness (%)**	83.2	53.5	59.3	67.7	58.6
*Number of observations*	*671*	*288*	*304*	*195*	*1086*
***Curative for adults***					
Quality score (%)	52.5	38.8	47.2	41.9	35.9
*Number of observations*	*667*	*288*	*287*	*181*	*831*
***Curative care for adults with diabetes***					
Quality score (%)	32.3	24.4	30.9	27.7	20.5
*Number of observations*	*652*	*162*	*241*	*153*	*277*
***Curative care for children***					
Quality score (%)	61.4	51.8	56.6	52.3	47.1
*Number of observations*	*666*	*285*	*272*	*174*	*917*
***Prenatal care***					
Quality score (%)	48.7	43.9	32.6	35.2	40.1
*Number of observations*	*657*	*238*	*86*	*115*	*816*
***All vignettes***					
Quality score (%)	48.8	41.4	44.7	40.1	39.3
*Number of observations*	*670*	*288*	*287*	*191*	*1082*

Crude associations between facility readiness scores and community SES quintiles, location, islands and provider type are presented in Table 3. Inequalities in readiness scores were the greatest for pustus, where there was a 13 percentage-point difference in readiness scores between facilities located in quintile 1 communities and those located in quintile 5 communities, where the mean readiness score was the highest. Regarding the urban and rural divide, puskesmas, pustus and midwife/nurse practices located in urban areas were better equipped; this was especially the case for pustus. There was also substantial variation between islands; the readiness scores were generally highest in Java islands across all facility types. The biggest difference was seen between puskesmas located in Central Java and Sumatra, with an 11-percentage point difference in readiness scores. Private facilities that provided services to JKN patients had higher readiness scores than those that did not.

**Table 3:** Association between readiness scores and community quintile, location, islands, and provider type, by facility type

	Public sector				Private sector					
	Puskesmas *N* = 671		Pustus *N* = 288		GP practices *N* = 304		Private clinics *N* = 195		Midwife/nurse practices *N* = 1086	
**Community SES quintile**	Score	95% CI	Score	95% CI	Score	95% CI	Score	95% CI	Score	95% CI
Poorest	83.7	(81.0–84.5)	47.4	(44.5–50.4)	61.9	(58.6–65.1)	65.6	(57.7–735)	57.8	(56.5–59.1)
Poorer	84.0	(82.4–85.5)	49.0	(46.3–51.7)	61.6	(58.5–65.6)	74.4	(69.6–81.3)	56.9	(55.8–58.5)
Middle	84.5	(82.8–86.2)	53.6	(51.3–56.0)	59	(56.6–61.8)	71.1	(67.3–74.5)	59.4	(57.6–60.4)
Richer	84.7	(83.0–86.4)	56.7	(53.5–59.8)	59.2	(56.9–60.9)	64.6	(61.0–67.8)	59.7	(58.4–61.2)
Richest	80.9	(79.0–82.9)	61.1	(58.0–64.2)	57.4	(54.8–59.2)	65.6	(62.6–68.6)	60.7	(59.0–62.2)
Equity difference (Q5-Q1)	−1.8		12.6***		−4.4*		0.0		3.1**	
**Type of location**										
Urban	84.2	(83.3–85.1)	56.7	(54.9–58.4)	58.6	(57.4–59.9)	67.3	(65.5–69.1)	59.5	(58.7–60.2)
Rural	80.2	(78.7–81.9)	48.5	(48.7–58.3)	63.8	(61.2–66.5)	70.7	(63.4–77.9)	57.4	(56.4–58.4)
Difference	4.0***		7.9***		−4.8**		−3.4		−2.1**	
**Island**										
Sumatra	78.1	(76.2–80.0)	50.4	(48.3–52.5)	65.3	(61.8–68.8)	70.9	(67.7–74.2)	59.9	(58.7–61.2)
West Java	80.1	(78.4–81.7)	60.1	(57.4–62.9)	57.9	(56.0–59.8)	64.4	(61.9–67.0)	61.6	(60.4–62.8)
Central Java	89.7	(88.8–90.7)	59.8	(55.8–63.9)	57.9	(55.6–60.2)	70.4	(65.5–75.3)	59.8	(58.4–61.2)
East Java	87.1	(85.4–88.7)	55.4	(53.2–57.6)	56.8	(53.3–60.4)	77.5	(70.8–84.2)	60.1	(58.8–61.4)
Lesser Sunda Islands	80.1	(77.5–82.8)	46.9	(43.6–50.2)	60.3	(56.8–63.9)	71.0	(42.2–99.8)	51.7	(49.5–54.0)
Kalimantan	86.3	(83.5–89.1)	49.4	(43.4–55.4)	61.9	(51.4–72.5)	76.8	(0–100)	56.7	(54.0–59.5)
Sulawesi	82.3	(79.5–85.2)	49.6	(44.5–54.7)	63.8	(58.8–69.0)	66.9	(56.7–77.2)	55.2	(52.1–58.3)
**KN providers**										
yes	83.1	(82.3–83.9)	54.1	(52.7–54.6)	65.6	(63.1–66.1)	73.5	(70.5–76.5)	63.3	(62.4–64.2)
no	88.1	(84.7–91.4)	48.7	(45.1–52.3)	57.6	(56.4–58.9)	65.7	(63.6–67.8)	57.0	(56.2–57.7)
Difference	−5.0*		5.6*		8.0***		7.8***		6.3***	

*p < 0.05, **p < 0.01, ***p < 0.001

Crude associations between facility knowledge scores and community SES quintile, location, island and provider type are presented in Table 4. There was a slight inequality in the knowledge score with respect to community SES and location of puskemas, where those located in Q5 and in urban areas had on average better knowledge scores. There was no inequality in knowledge scores with respect to community SES and location for the other types of facilities. However, variations existed across islands, with the Java islands performing best in terms of knowledge scores. GP and midwife/nurse practices that provided services to JKN patients had on average higher knowledge scores than those who did not.

**Table 4:** Association between knowledge scores and community quintile, location, islands, and provider type, by facility type

	Public sector				Private sector					
	Puskesmas *N* = 671		Pustus *N* = 288		GP practice *N* = 304		Private clinics *N* = 195		Midwife/nurse practice *N* = 1086	
**Community SES quintile**	Score	95% CI	Score	95% CI	Score	95% CI	Score	95% CI	Score	95% CI
Poorest	46.7	(44.7–48.8)	38.8	(34.3–43.4)	46.6	(42.3–52.5)	37.1	(26.5–44.7)	38.8	(37.2–40.8)
Poorer	49.0	(46.6–51.4)	41.1	(36.1–44.1)	48.6	(46.3–55.5)	48.7	(40.3–52.6)	38.9	(37.0–41.0)
Middle	47.0	(44.8–49.5)	39.2	(37.4–43.8)	46.4	(41.4–48.9)	42.7	(38.7–49.5)	40.0	(37.6–42.1)
Richer	50.0	(47.5–52.1)	46.1	(42.0–48.8)	41.5	(37.2–45.7)	35.6	(31.7–40.2)	40.5	(38.5–42.4)
Richest	50.9	(48.8–53.2)	40.1	(36.6–44.9)	42.8	(38.8–45.9)	38.3	(34.5–42.8)	39.9	(36.5–40.9)
Equity difference (Q5-Q1)	4.2**		1.2		−3.8		1.2		1.1	
**Type of location**										
Urban	49.7	(48.5–50.9)	41.9	(39.8–44.0)	44.2	(42.2–46.2)	40.5	(38.3–43.3)	39.9	(38.7–41.2)
Rural	46.1	(44.2–48.1)	40.4	(37.6–43.2)	48.3	(43.4–53.1)	35.9	(27.8–39.7)	38.6	(37.2–40.0)
Difference	3.6*		1.5		−4.1		4.6		1.3	
**Island**										
Sumatra	44.3	(42.3–46.4)	35.1	(32.5–37.8)	42.8	(38.5–47.1)	35.3	(31.0–39.6)	34.5	(33.0–36.1)
West Java	52.6	(50.8–54.5)	44.7	(41.5–48.0)	41.2	(38.1–44.3)	40	(36.8–42.8)	42.3	(40.3–44.4)
Central Java	52.5	(50.2–54.9)	48.2	(42.7–53.8)	50.2	(45.7–54.8)	48.9	(43.4–54.4)	47.1	(44.8–49.5)
East Java	45.3	(43.6–47.0)	38.6	(34.7–42.7)	46.0	(42.1–50.0)	33.9	(28.2–40)	37.6	(35.7–39.5)
Lesser Sunda Islands	43.8	(40.3–47.4)	41.3	(36.7–47.0)	46.0	(39.9–52.3)	38.7	(16.9–60.5)	37.1	(34.1–40.1)
Kalimantan	46.4	(41.5–51.5)	46.1	(38.1–54.0)	57.3	(40.1–74.5)	57.0	(0–100)	41.3	(38.1–44.6)
Sulawesi	43.7	(38.8–48.5)	29.2	(23.3–35.2)	41.0	(32.4–50.0)	38.0	(19.2–56.8)	32.6	(28.8–36.4)
**JKN providers**										
yes	48.5	(47.7–49.7)	41.6	(39.9–43.2)	46.8	(43.8–52.2)	44.6	(40.2–48.3)	43.6	(41.4–44.7)
no	50.5	(46.6–57.6)	39.6	(33.6–46.0)	44.6	(41.9–46.0)	38.1	(35.6–41.2)	38.1	(37.1–39.2)
Difference	−2.0		2.0		2.2		6.5**		5.5***	

*p < 0.05, **p < 0.01, ***p < 0.001

In order to understand whether the observed inequalities persisted when controlling for the combined effects of all covariates, regressions models for readiness and knowledge scores are presented in Table 5. In public facilities, we found a nonlinear, small but significant association between readiness scores and community SES. Public facilities located in quintile 3 and 4 communities had on average a 3.1 and 3.9 percentage point higher readiness score compared to facilities located in quintile 1 communities, respectively. Public facilities located in rural areas had readiness scores that were on average 4-percentage points lower than those located in urban areas. There were also disparities across islands, where facilities located in West Java, Sumatra, Lesser Sunda Islands and Sulawesi had significantly lower readiness scores compared to facilities located in Central Java, where the mean readiness score was the highest. In terms of knowledge scores, we did not find significant inequalities across SES groups or across urban and rural areas. Instead, we found that disparities remained across islands, with facilities located in East Java, Sumatra, Lesser Sunda Islands and Sulawesi having on average a lower knowledge score compared to facilities located in Central Java, where the mean knowledge score was the highest.

**Table 5:** OLS regressions for readiness and knowledge scores, by sector

	Public facilities		Private facilities	
	Readiness score	Vignette score	Readiness score	Vignette score
**Community SES quintile**				
Quintile 1	–	–	–	–
Quintile 2	1.1 (1.1)	2.0 (1.8)	−0.8 (1.1)	1.5 (1.8)
Quintile 3	3.1 (1.2)**	0.4 (1.5)	0.5 (1.0)	1.4 (1.7)
Quintile 4	3.9 (1.3)**	2.1 (2.8)	0.9 (1.2)	−1.2 (1.9)
Quintile 5	1.5 (1.5)	1.6 (1.7)	0.1 (1.2)	−2.3 (1.7)
**Location**				
rural	−4.3 (0.8)***	−0.21 (1.1)	−0.9 (0.9)	0.14 (1.4)
**Provider type (public)**				
Puskemas	–			
Pustu	−28.1 (1.0)***	−7.7 (1.2)***		
**Provider type (private)**				
Private physician	–	–	–	
Private clinics	–	–	7.2 (1.2)***	−4.9 (1.9)*
Midwife	–	–	−0.2 (0.8)	−8.3 (1.5)***
**JKN provider**				
yes	0.8 (1.5)	−1.4 (2.1)	7.1 (0.6)***	4.1 (1.0)***
**Island**				
Central Java	–		–	–
West Java	−6.4 (1.3)***	−0.6 (1.6)	0.6 (0.8)	−5.3 (1.8)**
East Java	−0.7 (1.1)	−7.1 (1.7)***	0.1 (0.8)	−8.4 (1.7)***
Sumatra	−8.8 (1.1)***	−9.6 (1.6)***	1.4 (0.9)	−11.0 (1.8)***
Lesser Sunda Islands	−9.4 (1.3)***	−7.2 (2.1)***	−6.3 (1.5)***	− 7.9 (2.5)**
Kalimantan	−2.5 (1.2)*	−4.4 (2.7)	−2.0 (1.4)	−3.8 (2.5)
Sulawesi	−5.5 (1.9)**	−11.2 (2.4)***	−1.9 (1.2)	− 11.7 (2.7)***
*Number of observations*	957	956	1584	1559
*Vignettes dummies*	NA	yes	NA	yes
*R square*	0.63	0.16	0.18	0.14

*p < 0.05, **p < 0.01, ***p < 0.001

Standard errors are in parentheses

Among the private health facilities, there was no evidence of inequalities in readiness or knowledge scores with respect to SES but there were large geographical differences across islands. The highest variation was observed for facilities located in West Java, East Java, Sumatra, Lesser Sunda Islands and Sulawesi where there was a 4 to 11 percentage point difference in average knowledge scores compared with facilities located in Central Java, which scored most highly. We also found that private facilities providing services to JKN patients had better readiness and knowledge scores that those that did not. Results from the regression models using the sub-domains of readiness are presented in Appendix 3.

## Discussion

Coverage is an important but insufficient goal for achieving a high quality health system as defined by the Lancet Commission [17]. Ensuring the availability of quality health care to everyone, irrespective of socioeconomic status, is a necessary condition for Universal health coverage (UHC). This goal is particularly challenging in countries like Indonesia, where the large population is spread across a vast archipelago of more than 6000 inhabited islands. Results of this study suggest that inequalities in the quality of care exist across islands, where public and private facilities located in Central Java were more likely to meet basic standards of facility readiness and to have higher knowledge scores than facilities located in East Java, West Java, Sumatra, Sulawesi and Lesser Sunda islands. This is in line with previous findings showing that provinces outside the most populated islands of Java and Bali often suffer from shortages in trained health personnel and basic health facility equipment and essential drugs [30, 31]. This study also shows that inequalities in readiness scores, unlike knowledge scores, go beyond the provincial level and can be observed between urban and rural areas. This was particularly the case in public sector facilities, where we found that urban location was a strong determinant of facility readiness: both puskesmas and pustus located in rural areas were more likely to have lower readiness scores than in urban areas. This result is in line with a recent World Bank survey, which found that beyond the island divide, significant disparities exist between rural and urban areas, with facilities located in urban areas performing better on the service-readiness and service availability than rural facilities [33].

The novelty of this paper lies in the analysis of inequalities beyond the geographical level and the rural/urban divide, by exploring the socio-economic inequalities in the readiness and clinical knowledge of primary health care facilities in Indonesia. We found some evidence that public facilities located in richer communities had slightly higher readiness scores than those located in poorer communities. However, the size of the effect was relatively small and was not significant for quintile 5 communities. Among private sector facilities, we did not find variation in either score across poorer and richer communities. However, we did find that higher-level and better-equipped private facilities, such as private clinics, were more often located in richer areas.

Among studies in other low- and middle-income countries (LMICs) that used clinical competence as a measure of quality, all found a correlation between provider competence and SES of the catchment area. Two studies from India linked households from two regions (Madhya Pradesh and Delhi) with a census of private and public providers in the same villages and found that in Madhya Pradesh, higher village SES was positively associated with greater numbers of health care providers and better public and private provider competence [11]. In Delhi, similar results were found, as moving from the richest to the poorest neighbourhoods was associated with a decrease in the clinical competence of providers [9]. In Tanzania, a study conducted in the Arusha region found that the competence of doctors in both private and public sectors was significantly lower in poorer regions [18]. One study conducted in the Democratic Republic of Congo found that women with lower socio-economic status lived in areas where the quality of care available was low compared to women with higher SES [14]. Two studies looked at the effect of pay-for-performance (P4P) schemes on inequalities in the performance of providers in Tanzania and Brazil. Prior to the introduction of the P4P scheme, both studies reported lower quality in deprived areas compared to richer areas, but these differences narrowed over time [5, 16]. In Indonesia, results from this study suggest that such inequalities in provider knowledge related to the area SES did not occur, which is encouraging. However, inequalities did persist across islands and across provider types.

Among the studies that used structural indicators to measure quality, evidence is mixed. Two studies conducted in Kenya linked population data with Service Provision Assessment Surveys [28]. One found that all quality metrics for maternal healthcare in public and private health facilities were lowest for the most impoverished areas and increased significantly with greater wealth [25]. The second one found little evidence of marked inequalities of inputs and service availability, although they did identify pro-rich inequalities in the availability of electricity, laboratory services, drug supply, and qualified staff in public health facilities [26]. The extent of inequalities found in these studies is greater than those reported in our study where inequalities in quality of care were primarily determined by the island and to a smaller extent the type of location (urban/rural) where Indonesians live.

This study also demonstrates that there is much still to be done to address quality of care across primary care in Indonesia. First, the items assessed in the facility readiness score and knowledge tested by the vignettes, can both be considered essential for the management of cases at this level, meaning that the low levels of readiness and knowledge scores is very worrying. Basic equipment, availability of essential medicines and diagnostic capacity were areas of key concern. The low readiness and knowledge scores found in midwife/nurse practices were particularly striking and in line with previous studies [3]. Second, we found that private facilities overall had worse scores than puskesmas, which is in line with the recent World Bank study, which found that on average, puskesmas had 6 extra components available compared to private GPs and clinics, and puskesmas outscored private clinics on all subdomains of general service readiness, with the difference most stark for diagnostic capacity [33]. In our study, we also found that puskesmas outscored private facilities on the basis of knowledge scores. Finally, we found that a key driver of readiness in private sector facilities (and to a lesser extent knowledge) was provider type, where facilities providing services to JKN patients had significantly higher readiness scores than those who did not. These results are in line with the Word Bank survey results, where facilities that were contracted by BPJS-Health were more likely to offer wider range of health services and have higher readiness scores than facilities that were not contracted [33].

Our findings have important implications in terms of access to and utilisation of health care services. With sizable user fees remaining in the private sector, equal availability certainly does not translate into equal access to quality care. In the public sector, the limited SES-related inequalities in quality of care are encouraging. However, it has been shown that out-of-pocket (OOP) payments are still incurred by patients in the public sector, even by members of the JKN [15]. The major cost drivers of OOP payments are medicines that patients purchase privately. Therefore, even in the public sector, low level of inequalities in availability of quality care will not necessarily translate into equal access and utilisation. A recent study showed that the effects of JKN on access and use of services were greater among people on low incomes and those in rural areas than among people on high incomes [1].

It is important to note that we focused on the notion of equality rather than equity. Equity implies distinguishing between “fair” and “unfair” sources of inequality. Inequalities can result from life choices, income, race, health status, as well as many other factors. While it seems reasonable to think that inequalities due to individual decisions will legitimately lead to inequalities in health utilisation, differences due to socio-economic factors should be avoided and considered illegitimate [6, 23]. Theoretically, as poorer populations might actually have greater health care needs, ensuring the principle of equity would lead to improving the quality of care in poorer areas specifically, and therefore reversing the imbalance created by what has been referred to the inverse care law, or the trend that “the availability of good medical care tends to vary inversely with the need of the population served” [27]. In this study, we show that even without considering the population’s needs, SES-related inequalities exist, although small in magnitude. It implies that the level of inequity might actually be higher than observed in this study, therefore deepening the gap between rich and poor in Indonesia.

Our study contains some limitations. Quality of care is a multidimensional concept. By focusing on facility readiness and knowledge scores, we did not capture other important aspects of quality such as patient satisfaction, clinical processes and health outcomes. Our measures of quality also had their own limitations. First, some recent studies have shown that structural quality is poorly correlated with process quality and health outcomes [19]. Second, the use of vignettes has been questioned due to the “know-do gap” documented in provider behaviour studies ([10] [21, 24];).. While careful interpretation is needed when using readiness and knowledge scores as proxies for “quality”, they are nonetheless important prerequisites to provide good quality care [33].

Another important limitation is the sampling strategy in this study. First, the IFLS is not representative of all Indonesian provinces, and therefore cannot produce a national estimate. IFLS 5 excluded most eastern Indonesian provinces, which are considered underdeveloped compared to their western counterparts, and where health facilities are often not even available [13]. The implication of this would be an underestimation of the extent of inequalities in both readiness and knowledge scores. Additionally, the facilities’ sampling frame was based on household responses to questions about known facilities in their local area. The list was not restricted to facilities that the respondents visited in order to limit any biases resulting from a choice-based sample. We cannot however, exclude the possibility that respondents are more likely to know about facilities they used.

### Policy implications

Since the launch of the JKN and since this data was collected, multiple initiatives have been adopted to improve the quality of care in Indonesia. Reforms focused on improving facilities’ infrastructure in deprived areas, increasing supply of drugs and revising guidelines and regulations to expand the role of primary health centres in health promotion and prevention [20]. The Ministry of Health has also set up a primary care accreditation commission (Komisi Akreditasi Fasilitas Kesehatan Tingkat Primer – KAFKTP) to improve quality of services by ensuring that the necessary inputs (such as infrastructure, equipment and human resources), clinical and managerial processes are in place. The commission also provides follow-up support to ensure continuous improvement and reaccreditation every 3 years. In 2018, BPJS-Health also implemented performance-based capitation that aims to measure the commitment of primary care providers to deliver primary care services comprehensively, based on the contact rate, percentage of chronic conditions visits, and non-specialised referral ratio.

The consequences of these reforms are twofold. First, by focusing on rural and deprived areas, these reforms represent a unique opportunity to improve quality of care in Indonesia, and to redress the current inequalities between major islands, rural and urban areas, and to a lesser extent between deprived and richer areas. Second, as we found that private providers contracted by BPJS tend to offer better quality of care, encouraging private providers to join the JKN program might improve access to quality care in this context. Private providers need to meet minimum criteria set by the BPJS-Health to be contracted and the receipt of the capitation payment from BPJS-Health has been shown to improve the service readiness of the contracted private facilities [33]. Engaging with private facilities to join the JKN program is a unique opportunity to potentially improve quality in the private sector, either through initial standards for joining the JKN or by encouraging private facilities to use their capitation fees for quality improvement.

## Conclusion

As the policy landscape is changing in Indonesia, measurement of inequalities in quality of care is needed to monitor progress to UHC. In this study, we found that inequalities in facilities’ readiness exist across major islands in Indonesia, across rural and urban areas for public sector facilities, and to a small but non-negligible extent across poorer and richer communities for public sector facilities. As cost barriers affect the poorest individuals, ensuring that all communities have access to well-equipped health facilities with competent providers is a minimum necessity for achieving UHC.

## Supplementary Information

**Additional file 1.**

**Additional file 2.**

**Additional file 3.**

## Data Availability

The IFLS data that support the findings of this study are available from RAND https://www.rand.org/well-being/social-and-behavioral-policy/data/FLS/IFLS/ifls5.html

## Abbreviations

SES: Socio-economic status
MMR: Maternal mortality ratio
UHC: Universal health coverage
LMIC: Low- and middle-income country
P4P: Pay for performance
JKN: Jaminan Kesehatan Nasional
IFLS: Indonesian family life survey
GP: General practitioner
SARA: Service availability and readiness assessment
OOP: Out-of pocket

## Footnotes

1 We ran a robustness test by including ‘province’ fixed effects instead of ‘island’ fixed effects. Results were unchanged and therefore not shown.
