# Supplementary material for: Poor quality for the poor? A study of inequalities in service readiness and provider knowledge in Indonesian primary health care facilities
Source: Int J Equity Health. 2021 Nov 4;20:239. doi: 10.1186/s12939-021-01577-1 (PMC8567576; doi:10.1186/s12939-021-01577-1)
Supplement: Supplementary file 1 — Additional file 1. [file 12939_2021_1577_MOESM1_ESM.docx]

Appendix 1: Indicators for general service readiness used in analysis

| *Domains* | *Indicators* |
| --- | --- |
| **Basic amenities (8)** | Power, internal water source, curtain to close of examination room (observed), clean examination floor and walls (observed), running water to wash hands in the examination room (observed), garbage can in the examination room (observed), examination table (observed), toilet facilities |
| **Infection prevention (4)** | Sterilisation/autoclaves, alcohol, Betadin, gloves |
| **Basic equipment (17)** | Regular stethoscope, stethoscope for pregnant mothers, blood pressure meter, adult scale, infant scale, thermometer, measure for body height, communication equipment.  **Lab specific:** Sahli set, giemsa stain solution, benedict solution, wright solution, strips for pregnancy test, urine protein test strips, urine glucose test strips, microscope, centrifuge |
| **Essential medicines (15)** | Oral antibiotic, eye antibiotic, analgesic, antipyretic, anti-fungal, anthelmintics, anti-TB, anti-malarial, ORS, iron tablets, vitamin A, medicine for BP, anesthetic, medicine for cholesterol, medicine for blood sugar |
| **Diagnostic capacity (8)** | Haemoglobin, leucocyte estimation, blood type estimation, erythrocyte estimation, urine analysis, pregnancy test, faeces examination, sputum examination |

Notes: All indicators were coded as 1 if the interviewee reported the presence of the items. If not, the indicator was recoded as 0.
