# Supplementary material for: Poor quality for the poor? A study of inequalities in service readiness and provider knowledge in Indonesian primary health care facilities
Source: Int J Equity Health. 2021 Nov 4;20:239. doi: 10.1186/s12939-021-01577-1 (PMC8567576; doi:10.1186/s12939-021-01577-1)
Supplement: Supplementary file 2 — Additional file 2. [file 12939_2021_1577_MOESM2_ESM.docx]

Appendix 2: Details on the criteria used in the medical vignettes

For prenatal care, nineteen criteria were identified based on international guidelines for routine pregnancies (Villar & Bergsjo, 1997). For the adult presenting with cough and fever, eleven criteria corresponding with guidelines for the integrated management of adult illness for environments of high tuberculosis prevalence were selected (World Health Organization, 2004). For the scenario of a child with diarrhea and vomiting, the twelve criteria were coded against guidelines for the integrated management of childhood illnesses (World Health Organization, 2002). For the scenario of an adult with diabetes, the IFLS indicated the criteria to include but did not mention the guideline used.

| Prenatal case | Adult curative care | Child curative care | Adult care with diabetes |
| --- | --- | --- | --- |
| **Evaluate hypertensive disorders**  1. Ask history of high blood pressure  2. Take blood pressure  3. Test urine protein  4. Ask about smoking habit  **Take History and Physical**  5. Ask about history of heart disease  6. Ask about history of diabetes  7. Ask about family history of illnesses  8. Take height measurements  9. Weigh patient  10. Measure uterine height  11. Assess whether high-risk pregnancy  **Perform diagnostics and prevention**  12. Determine tetanus  immunization status  13. Test for sexually transmitted infections  14. Test hemoglobin levels  15. Advise on nutrition  16. Give iron-folate  **Establish care management**  **system**  17. Date the pregnancy  18. Plan for delivery  19. Plan for follow-up visits | **Take history**  1. Ask about duration of illness  2. Ask about previous  respiratory illness  3. Ask about blood in cough  4. Ask about color of  sputum  5. Ask about chest pain  **Conduct physical, sputum**  6. Take temperature  7. Listen to respiration  8. Examine throat  9. Assess chest in drawing  10. Assess for cyanosis  11. Test sputum | **Take history**  1. Ask about duration of illness  2. Ask about frequency of illness  3. Ask about appearance of stools/vomit  4. Ask about blood in  stools  5. Ask about fever  **Conduct physical**  6. Take temperature  7. Check for sunken  fontanelles  8. Check skin turgor  9. Take pulse  10. Check alertness  **Provide care and advice**  11. Administer oral  rehydration fluids  12. Recommend when to return if worse | **Questions about present condition**  1- Ask about duration of illness  2-Ask about history of medication  3-Ask about frequency of urine  4- Ask about frequency of thirst  5- Ask about weigh loss  6- Ask about sweating  7-Ask about anxiety and heart palpitations  8- Ask about abdominal fullness after meals  9-Ask about edema or weigh retention  10-Ask about current treatment for hypertension  11-Ask about tingling feeling  12- Ask about wound that stays  13- Ask about ulcer  14- Ask about family history  15- Ask about weary feeling  16- Ask about blood sugar check  **Take history**  17-History of hypertension?  18- History of high cholesterol?  19-Co-existing or prior heart condition?  20-Prior eye examination?  21-Prior hospitalization?  22-Prior diabetic coma?  23-Prior renal failure?  24-Does he smoke regularly?  25- Number of packages/quantity of smoking?  26-Alcohol use?  27-Immunization history?  28-Regular exercise?  29-Questions about nutrition/eating habits?  30-Is there any family member with this disease?  **Physical examination**  31-Blood pressure in one arm  32-Blood pressure in both arms  33-Listen to chest/heart?  34-Listen to abdomen?  35-Examine the feet?  36-Examine peripheral vascular system?  37-Check for edema?  38-Examine prostate?  39-Pulse  40-Respiration  **Laboratory exams**  41-Blood chemistry: creatinine, glucose?  42-Sputum exam?  CBC (Complete Blood Count)?  43-Test for triglycerides?  44-Liver function?  45-HgbA1c?  46-Hepatic enzymes? |
