# Supplementary material for: Poor quality for the poor? A study of inequalities in service readiness and provider knowledge in Indonesian primary health care facilities
Source: Int J Equity Health. 2021 Nov 4;20:239. doi: 10.1186/s12939-021-01577-1 (PMC8567576; doi:10.1186/s12939-021-01577-1)
Supplement: Supplementary file 3 — Additional file 3. [file 12939_2021_1577_MOESM3_ESM.docx]

Appendix 3: OLS regressions obtained with the subdomains of readiness score

|  | **Public facilities** | | | | | **Private facilities** | | | | |
| --- | --- | --- | --- | --- | --- | --- | --- | --- | --- | --- |
|  | Basic amenities | Basic equipment | Infection prevention | Diagnostic capacity | Essential medicine | Basic amenities | Basic equipment | Infection prevention | Diagnostic capacity | Essential medicine |
|  |  |  |  |  |  |  |  |  |  |  |
| **Community SES quintile** |  |  |  |  |  |  |  |  |  |  |
| Quintile 1 | - | - | - | - | - | - | - | - | - | - |
| Quintile 2 | -0.1 (1.8) | 2.0 (1.8) | -0.5 (1.4) | 3.7 (2.7) | 0.4 (1.7) | -0.4 (1.4) | 0.08 (1.6) | 2.0 (1.3) | 0.0 (1.5) | -2.9 (1.6) |
| Quintile 3 | 2.9 (2.0) | 3.0 (1.8) | 1.1 (1.4) | 8.1 (2.9)*** | 0.5 (1.6) | 2.1 (1.4) | 2.6 (1.4) | 0.5 (1.5) | -0.6 (1.5) | -2.1 (1.5) |
| Quintile 4 | 3.1 (1.9) | 4.0 (2.0)* | 1.5 (1.4) | 7.4 (3.4)* | 3.8 (1.7)* | 0.3 (1.3) | 2.8 (1.7) | 3.9 (1.4)** | -1.5 (1.7) | -1.1 (1.5) |
| Quintile 5 | 2.2 (1.9) | -1.4 (2.2) | 2.6 (1.5) | 2.1 (3.7) | 2.2 (1.7) | 0.7 (1.3) | 2.4 (1.7) | 2.7 (1.6) | -1.6 (1.8) | -3.4 (1.7)* |
| **Location** |  |  |  |  |  |  |  |  |  |  |
| rural | -5.3 (1.3)*** | -4.7 (2.0)*** | -3.1 (1.0)*** | -6.6 (2.1)** | -2.0 (1.2) | -4.3 (1.0)*** | -1.2 (1.2) | -2.7 (1.0)** | 0.22 (1.3) | 3.4 (1.1)** |
| **Provider type (public)** |  |  |  |  |  |  |  |  |  |  |
| Puskemas | - | - | - | - | - |  |  |  |  |  |
| Pustu | -14.3 (1.4)*** | -37.2 (1.4)*** | -14.5 (1.4)*** | -53.3 (2.1)*** | -21.6 (1.2)*** |  |  |  |  |  |
| **Provider type (private)** |  |  |  |  |  |  |  |  |  |  |
| Private physician |  |  |  |  |  | - | - | - | - | - |
| Private clinics |  |  |  |  |  | -1.3 (1.2) | 12.6 (1.8)*** | 7.7 (1.5)*** | 16.6 (2.8)*** | 0.5 (1.5) |
| Midwife |  |  |  |  |  | -0.4 (0.9) | 7.6 (1.3)*** | 4.6 (1.2)*** | 1.2 (1.5) | -14.1 (1.3)*** |
| **JKN provider** |  |  |  |  |  |  |  |  |  |  |
| yes | 4.2 (2.6) | 0.5 (2.1) | 2.1 (1.9) | -6.9 (3.7) | 4.1 (1.8)* | 3.1 (0.8)*** | .11.3 (0.9)*** | 6.3 (0.8)*** | 8.2 (1.2)*** | 6.4 (1.0)*** |
| **Island** |  |  |  |  |  |  |  |  |  |  |
| Central Java | - | - | - | - | - | - | - | - | - | - |
| West Java | -2.1 (1.5) | -8.8 (1.7)*** | 1.8 (1.4) | -23.4 (3.1)*** | 0.5 (1.4) | -0.7 (0.8) | -0.5 (1.2) | -1.7 (1.1) | 0.0 (1.5) | 5.9 (1.5)*** |
| East Java | 0.3 (1.7) | 0.2 (1.5) | -0.6 (1.3) | -3.8 (3.2) | 1.1 (1.5) | -1.0 (1.2) | -1.4 (1.2) | -0.4 (1.2) | 0.4 (1.4) | 3.1 (1.5)* |
| Sumatra | -7.5 (1.8)*** | -10.4 (1.9)*** | -0.7 (1.3) | -22.3 (3.1)*** | -2.9 (1.6) | -0.5 (1.2) | 0.3 (1.4) | -2.1 (1.0)* | 1.0 (1.5) | 8.8 (1.5)*** |
| Lesser Sunda Islands | -6.6 (1.1)*** | -11.3 (2.4)*** | -2.4 (1.7) | -16.3 (3.7)*** | -10.2 (2.2)*** | -6.4 (1.8)*** | -10.3 (2.1)*** | -6.7 (2.0)*** | -4.5 (1.9)* | -3.5 (1.8) |
| Kalimantan | 0.5 (2.2) | -4.5 (2.0)* | 0.3 (2.9) | -5.6 (3.4) | -2.3 (2.0) | -0.5 (1.8) | -4.6 (2.1)* | -3.8 (2.3) | -3.6 (1.8) | 2.6 (2.1) |
| Sulawesi | -10.0 (2.7)*** | -5.3 (3.0) | 0.5 (1.7) | -10.1 (4.4)* | -2.8 (1.9) | -3.2 (1.9) | -2.9 (1.8) | -8.7 (1.7)*** | 2.9 (1.7) | 2.3 (2.3) |
| ***Number of observations*** | **957** | **957** | **957** | **957** | **957** | **1584** | **1584** | **1584** | **1584** | **1584** |
| ***R square*** | **0.29** | **0.61** | **0.31** | **0.34** | **0.47** | **0.07** | **0.17** | **0.10** | **0.12** | **0.20** |

*p < 0.05, **p < 0.01, ***p < 0.001. Standard errors are in parentheses
